# The Identification and Quantification of 21 Antibacterial Substances by LC-MS/MS in Natural and Organic Liquid Fertilizer Samples

**DOI:** 10.3390/molecules29071644

**Published:** 2024-04-06

**Authors:** Ewelina Patyra, Zbigniew Osiński, Krzysztof Kwiatek

**Affiliations:** Department of Hygiene of Animal Feedingstuffs, National Veterinary Research Institute, 24-100 Pulawy, Poland; zbigniew.osinski@piwet.pulawy.pl (Z.O.); kwiatekk@piwet.pulawy.pl (K.K.)

**Keywords:** natural liquid fertilizers, organic liquid fertilizers, slurry, post-fermentation sludge, LC-MS/MS, antibiotics, method validation

## Abstract

Antibiotics in animal production are widely used around the world for therapeutic and preventive purposes, and in some countries, they still serve as antibiotic growth stimulants. Regardless of the purpose of using antibiotics in livestock, they may be present in animal tissues and organs as well as in body fluids and excretions (feces and urine). Farm animal excrement in unprocessed form (natural fertilizers) or processed form (organic fertilizers) is applied to agricultural fields because it improves soil fertility. Antibiotics present in fertilizers may therefore contaminate the soil, surface, groundwater, and plants, which may pose a threat to the environment, animals, and humans. Therefore, it is important to develop analytical methods that will allow for the control of the presence of antibacterial substances in natural and organic fertilizers. Therefore, in this study, an LC-MS/MS method was developed and validated for the determination of 21 antibacterial substances in natural and organic liquid fertilizers. The developed method was used to analyze 62 samples of natural and organic liquid fertilizers, showing that over 24% of the tested samples were contaminated with antibiotics, mainly from the group of tetracyclines and fluoroquinolones. Studies of post-fermentation sludge from biogas plants have shown that the processes of anaerobic methane fermentation, pH, and temperature changes taking place in bioreactors do not lead to the complete degradation of antibiotics present in the material used for biogas production. For this reason, monitoring studies of natural and organic fertilizers should be undertaken to limit the introduction of antibiotics into the natural environment.

## 1. Introduction

Increasing requirements in the field of food and feed safety and the natural environment mean that veterinary medicinal products, including antibacterial substances such as antibiotics, sulfonamides, and quinolones, should be subject to strict control. Their wide-scale use in veterinary medicine, especially in farm animals for therapeutic and non-therapeutic purposes, has resulted in a number of side effects, mainly related to the spread of pathogenic microorganisms resistant to the action of these drugs. In order to systematically ensure the health protection of animals and consumers consuming food of animal origin in European Union countries, including Poland, programs have been implemented to control, among others, antibacterial substances in manufactured medicated feeds and non-target feeds, as well as water from animal drinking systems, and a national residue control testing program was developed, the scope of which includes a total of over 280 compounds ranging from banned anabolic substances from group A (hormones, thyrostatic substances, etc.) for veterinary medicinal products and environmental pollutants (metals, pesticides, etc.) included in group B [1,2,3]. The control programs created did not take into account the environment of animal production, in particular farm animal excrement, which, in processed or unprocessed form, is applied to arable land or permanent grassland in the form of natural fertilizers or organic fertilizers. The use of these fertilizers is increasing because it makes it possible to utilize a significant number of products from the rendering sector and the livestock breeding environment. This translates into the ability to maintain appropriate biological, physical, and chemical properties of the soil, and thus directly affects the state of the natural environment, as well as the safety of food production of plant origin (e.g., cereals, vegetables, and fruits) and animal origin through feed. Currently, there is a lack of data on the presence and content of antibacterial substances in natural and organic fertilizers, which makes it impossible to estimate the amount of their transfer to the natural environment.

Natural fertilizers such as pig and cattle manure, poultry manure, and slurry are used all over the world to fertilize arable soils and permanent grasslands. In addition to natural fertilizers, organic fertilizers are used for crops, which are produced using animal by-products. Natural fertilizers and organic fertilizers, depending on their state of matter, can be divided into solid fertilizers (manure, poultry manure, granulated cattle manure, poultry manure, and horse manure) and liquid fertilizers, which include slurry and post-fermentation sludge from biogas plants, composting plants, bicomposting plants, biohumus, etc.

In accordance with Regulation (EC) No. 1069/2009 of the European Parliament and the Council of 21 October 2009, which defines the concept of organic fertilizers or soil improvers, these may be category 2 materials, which means that they may be products containing residues of approved substances or contaminants in amounts exceeding permitted levels, including antibiotics, sulfonamides, and quinolones, which should be monitored in live animals and products of animal origin [4].

The available literature data indicate that antibiotics are often detected in natural fertilizers, and their concentrations range from several micrograms per kilogram to several hundred milligrams per kilogram [5,6,7,8,9,10,11,12,13,14]. Scientific data also show that antibiotics are most often detected in the feces of pigs from large-scale farms [15]. However, the research most often concerns solid natural fertilizers such as pig feces or poultry manure [5,6,7,8,9,11]. Scientists are less likely to focus on the analysis of slurry (liquid manure) or organic fertilizers produced based on animal by-products, such as post-fermentation sludge from biogas plants, or commercial organic fertilizers. Slurry, by definition, is a mixture of feces, urine, and water used to clean livestock rooms. The slurry is stored in tanks appropriate for this purpose, so the antibiotics contained in animal excrements are partially diluted and degraded due to storage time, pH, or the presence of microorganisms. In the production of biogas, animal by-products are used, such as animal excrement, slaughterhouse waste, but also feed, and many others. The digestate produced after biogas production is used as organic fertilizer for arable fields and permanent agricultural land. Despite the technological processes used in biogas production, such as methane fermentation using appropriate microorganisms and changes in pH and temperature, the antibiotics present in the input used are not completely degraded. Currently, natural fertilizers are not subject to any laboratory control, while organic fertilizers that are introduced to the market are subject to registration and laboratory tests for the content of chemical (heavy metals) and biological contaminants (presence of *Salmonella* bacteria, the number of *Enterobacteriaceae*, *Escherichia coli*, the presence of live parasite eggs—*Ascaris* sp., *Trichuris* sp., *Toxocara* sp.) in Poland [16].

Frey et al. described the test results for samples of pig feces and slurry from 11 European countries, as well as America (Mexico and the USA) and Asia (China). In the described pig slurry samples, the most frequently used group of antibiotics were sulfonamides, followed by fluoroquinolones and tetracyclines in combination with other antibiotics. The maximum concentrations for detected antibiotics were up to 28,000 µg/kg [9]. Wallace and Aga described an analysis method enabling the detection of the presence of antibiotics from the tetracycline group, sulfonamides, and macrolides in pig slurry and feces. They found the presence of antibiotics both in slurry and pig feces, and the most frequently found compounds were antibiotics from the tetracycline group: tetracycline, 4-epi-tetracycline, chlortetracycline, and 4-epi-chlortetracycline, as well as oxytetracycline and anhydrotetracycline [12]. Research conducted by Patyra et al., who analyzed fourteen samples of post-fermentation sludge from biogas plants in Poland, found the presence of enrofloxacin and tiamulin in two samples, at concentrations of 50 µg/kg and 148 µg/kg, respectively. Moreover, the authors detected the presence of doxycycline in pig slurry in an amount of 5900 µg/kg [8]. Studies described by scientists from around the world regarding the analysis of feces and slurry of farm animals for the presence of veterinary drugs indicate that these drugs are very often present in the tested samples. This may pose a threat to the natural environment by introducing such fertilizers into agricultural fields and permanent grasslands [5,6,8]. Therefore, the aim of the presented work was to develop, validate, and analyze samples of natural liquid fertilizers (pig and cattle slurry) and organic fertilizers (digestate sludge from biogas plants and commercial organic fertilizers produced on the basis of animal by-products) using the LC-MS/MS technique to assess the presence of selected antibiotics used for medicinal purposes in animal production. The method presented in this work is one of the few methods available in the international literature that allows for the detection and determination of antibacterial substances in natural and organic liquid fertilizers. Moreover, we analyzed samples of post-fermentation sludge from biogas plants for antibacterial substances and proved that these fertilizers contained antibiotics. The research carried out can be considered pioneering research on a European scale.

## 2. Results

### 2.1. Method Development

To analyze antibacterial substances in animal feces and slurry, researchers use the liquid chromatography technique with different types of detection, depending on whether the method is developed for one chemical group of antibiotics or antibiotics from different chemical groups (multi-component method) [5,6,7,8,16,17,18,19]. In the case of analysis of a single group of compounds, liquid chromatography with UV detection is used (HPLC-UV) [6,19]. Hu et al. used a mobile phase consisting of oxalic acid and acetonitrile at a UV light wavelength of 278 nm to analyze tetracycline antibiotics. However, for selected antibacterial substances from the group of sulfonamides and quinolones, they used a mobile phase consisting of ammonium acetate in water and acetonitrile. They chose 265 nm as the optimal wavelength of UV light [19]. To analyze sulfonamides in natural fertilizers (liquid and solid), Osiński et al. used the HPLC technique but with fluorescence detection (HPLC-FLD). They used 0.08% acetic acid in water combined with acetonitrile and methanol as the mobile phase for the analysis of sulfonamides. For the separation of sulfonamides, they used a Zorbax Eclipse XDB C18 column (150 × 4.6 mm, 5 μm), [7]. However, liquid chromatography with mass spectrometry is most often used to analyze antibacterial substances in the feces and slurry of farm animals and post-fermentation sludge from biogas plants [5,8,10,12]. This is due to the fact that this method is more sensitive than UV or FLD detection and allows for the simultaneous analysis of several or several dozen antibacterial substances belonging to different chemical groups during one analysis. Rashid et al. (2020) developed an LC-MS/MS method for the analysis of forty antibacterial substances from various chemical groups in poultry and pig feces, as well as in poultry feed and soil samples [20]. Berendsen et al. used the LC-MS/MS technique to analyze 25 antibacterial substances in animal feces. The analytical method they developed allowed for the detection of compounds in the concentration range from 2.5 µg/kg to 10 µg/kg, depending on the compound being determined [5]. For the analysis of antibiotics using liquid chromatography, chromatographic columns with C18 octadecyl packing of various lengths and diameters, from different manufacturers, are most often used [5,6,7,8,11,21]. The most commonly used mobile phase for LC-MS analysis is formic acid in water or ammonium formate in water combined with acetonitrile or methanol with/or without the addition of formic acid or ammonium formate [5,6,7,8,11,21]. In our work, a mobile phase consisting of formic acid in water and formic acid in acetonitrile was used, and the separation of antibiotics was carried out on a Kinetex C18 column with a length of 75 mm and a diameter of 2.1 mm, which allowed for the satisfactory separation of twenty-one antibiotics belonging to six chemical groups in within 23 min.

Natural and organic liquid fertilizers have a pH close to neutral or alkaline. The pH measurements we performed showed that it ranged from 7.2 to 9.0. Therefore, in order to extract antibacterial substances belonging to various chemical groups from slurry and digestate sludge from biogas plants and commercial organic fertilizers, the first step is to measure the pH of the fertilizer and correct the pH to a value of 4. This step is necessary to enable the extraction of all analytes in one extraction protocol, in particular antibiotics from the tetracycline group, which are zwitterionic at pH = 4 and interact less frequently with natural organic matter. Wallace and Aga used glacial acetic acid to correct the pH of fertilizers [12]. In our work, we used 85% orthophosphoric acid to acidify samples of natural and organic liquid fertilizers to pH = 4.

The analysis of antibacterial substances from animal feces or slurry is difficult due to the large amount of organic matter contained in the sample. For this reason, to extract antibiotics, hormones, and antiparasitic drugs from fertilizer samples, scientists use, among others, sonication or pressurized liquid extraction [5,22,23,24]. However, the most common method is to shake samples using a laboratory shaker. To extract analytes belonging to various chemical groups, scientists most often use a McIlvaine–NA_2_EDTA buffer, EDTA solution, or a combination of McIlviane–Na_2_EDTA buffer with methanol or acetonitrile [5,8,11]. The McIlvaine–Na_2_EDTA buffer is prepared by mixing appropriate amounts of 0.1 M citric acid with 0.2 M disodium hydrogen phosphate and adding Na_2_EDTA, which has a chelating effect on metal ions present in the analyzed sample. However, the purification of the obtained extract is carried out using various techniques. For example, Argüeso-Mata et al. combined dispersive solid-phase extraction (d-SPE) and compact solid-phase extraction (c-SPE) to extract 21 analytes from different groups such as tetracyclines, macrolides, sulfonamides, β-lactams, and fluoroquinolones [25]. The scientific literature also proposes the use of the QuEChERS technique [26] and normal solid-phase extraction using cartridges [16]. For the analysis of 29 antibacterial and antiparasitic substances, Nebot et al. proposed extraction using the MeOH–McIlvaine–EDTA mixture, 70:30, *v*/*v*, and then filtering the obtained extract through a syringe filter and HPLC-MS/MS analysis [24]. However, solid-phase extraction using polymeric cartridges such as Strata-X or Oasis HLB is most often used to purify and concentrate the extract [5,10,11,12,19].

**Figure 1:**
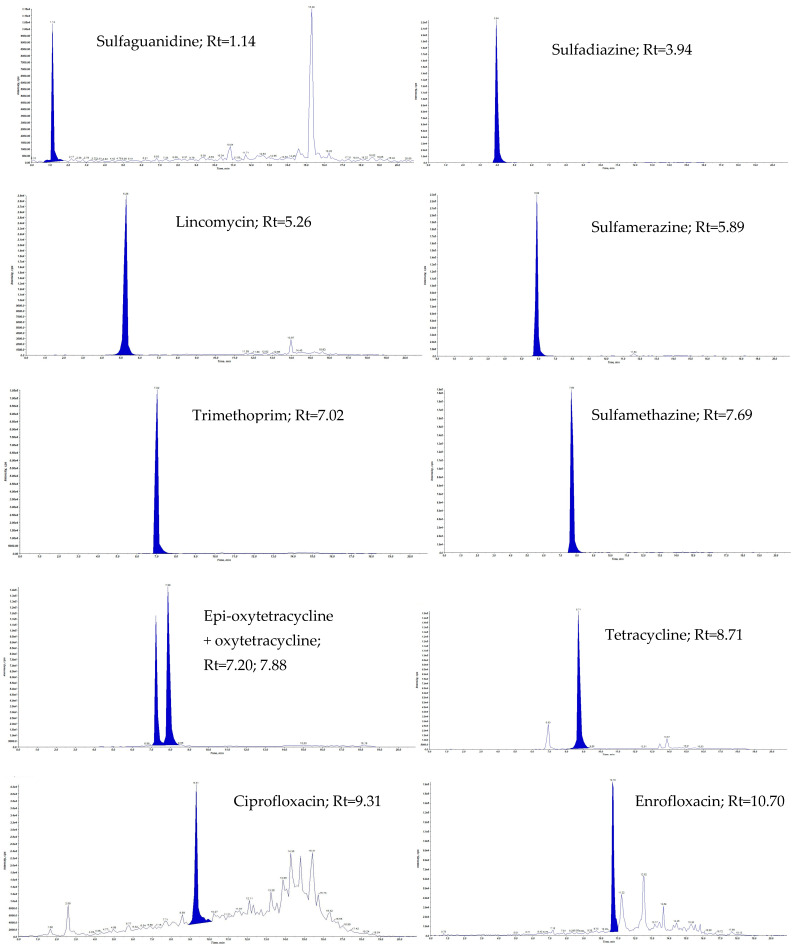

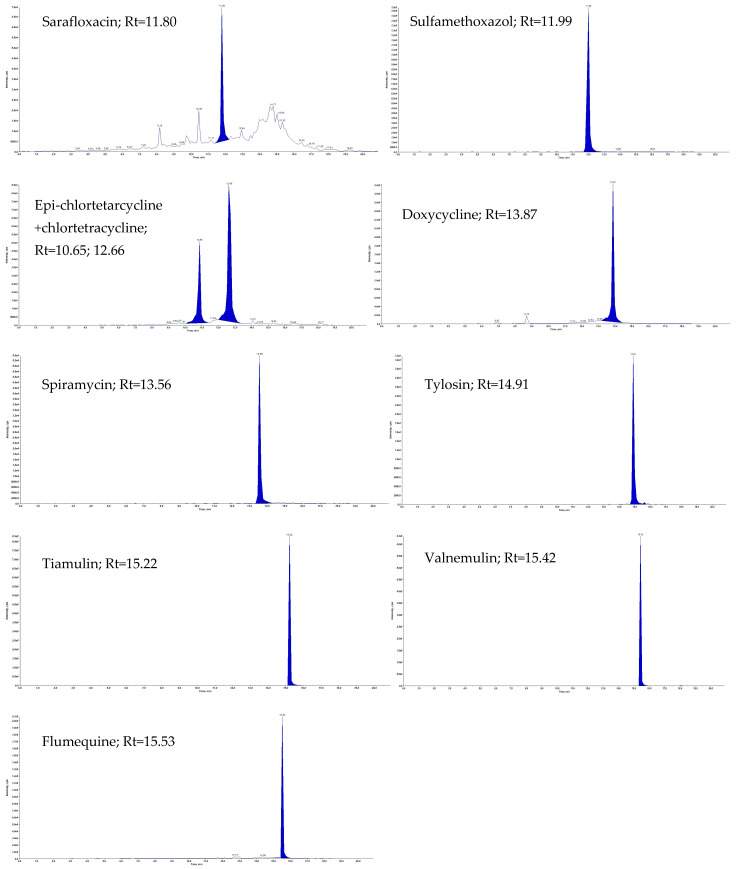
MRM chromatograms of liquid-manure-sample-fortified 21 antibacterial substances at the concentration level 10 μg/kg and 20 μg/kg for ciprofloxacin, enrofloxacin, and sarafloxacin.

In our work, in the first stage, we used the method described by Patyra et al., who used a McIlvain–Na2EDTA buffer for the extraction of lincomycin, tiamulin, tylosin, trimethoprim, oxytetracycline, tetracycline, chlortetracycline, doxycycline, enrofloxacin, and ciprofloxacin, and double purification of the extract using d-SPE and then SPE on Strata X-CW cartridges [8]. Unfortunately, this approach was not beneficial for all 21 analytes. Therefore, for natural and organic liquid fertilizers, another solution had to be found that would enable the extraction and purification of 21 antibacterial substances belonging to six chemical classes. The most advantageous solution turned out to be the use of a mixture of acetonitrile, methanol, and McIlvaine–Na2EDTA buffer in the proportion of 3.75: 1.25: 3 *v*/*v*/*v* for 5 g samples of natural and organic liquid fertilizers. After shaking and centrifugation, 6 mL of the obtained extract was diluted in 25 mL of water to reduce the content of organic solutions, and purification was performed on Strata-X polymer cartridges. The obtained eluate was evaporated to dryness in a stream of nitrogen, and the precipitate was dissolved in 500 µL of 0.1% formic acid in water and additionally filtered through a 0.22 µm PVDF filter into an amber chromatography vial and analyzed by LC-MS/MS. Multiple reaction monitoring (MRM) chromatograms of liquid-manure-sample-fortified 21 antibacterial substances at the concentration level 10 µg/kg and 20 µg/kg (for ciprofloxacin, enrofloxacin, and sarafloxacin) are presented in Figure 1.

### 2.2. Validation of the Method

The developed analytical method was validated in accordance with the EU Commission Implementing Regulation 2021/808 [27]. Validation parameters such as linearity, repeatability, intralaboratory reproducibility, recovery, selectivity, the limit of detection (LOD), the limit of quantification (LOQ), decision limit (CCα), and detection capability (CCβ) were assessed, and the uncertainty (U,%) for each analyte was estimated. On each validation day, a matrix calibration curve was plotted that included seven points from 0 to 500 µg/kg or 0 to 750 µg/kg for CIP, ENR, and SAR. The coefficient of determination (R^2^) obtained for each compound was above 0.98 or more, indicating the good linearity of the method. The coefficient of variation (CV, %) for repeatability ranged from 5.3% to 18.0%, while the CV (%) for intralaboratory reproducibility ranged from 7.6% to 20.1% and was the highest for sulfaguanidine. The recovery for all the analyzed antibiotics ranged from 88.6% to 114.6%. The determined LOD and LOQ values ranged from 2.9 to 13.1 µg/kg and from 5.1 to 18.2 µg/kg, respectively. The calculated worst-case uncertainty value was 36% for enrofloxacin. The validation parameters obtained comply with the values considered acceptable by this regulation. All validation parameters of the method are presented in Table 1.

### 2.3. Real-Sample Analysis

Laboratory tests of pig and cow slurry and organic fertilizers (fermentation sludge from biogas plants and sludge from composting and biocomposting plants and the processing plant) based on the LC-MS/MS technique confirmed the presence of antibacterial substances. The tests carried out showed the presence of antibacterial substances in 14 out of 62 analyzed samples of sludge from biogas plants, composting plants, biocomposting plants, and processing plants, which constituted over 24.4%. The determined levels of antibacterial substances in organic fertilizers ranged from 13.8 µg/kg to 1002.0 µg/kg. The obtained results may indicate that the applied processing processes, despite the change in pH input, temperature increase during processing or composting, and the action of microflora conducting methane fermentation, did not lead to the complete degradation of antibacterial substances present in the material used. The most frequently found antibiotics in post-fermentation sediments were ENR, as well as ENR and CIP, which is a metabolite of ENR produced in a living organism. The presence of ENR may indicate that the input used to produce the fertilizer contained poultry manure or cattle feces. ENR is permitted for use in these animal species. When treating poultry, ENR can be used in drinking water (except for laying hens), while in cattle, it can be used to treat calves by dissolving it in water or milk. In addition to those mentioned, the presence of DC in four samples; TIAM in three samples; and the presence of TYL, LINCO, and OXT was confirmed in organic fertilizers. An interesting case was a sample of liquid digestate from a biogas plant from one voivodeship in Poland. This sample contained as many as seven different antibacterial substances, namely LINCO, TIAM, TYL, CIP, ENR, DC, and OXT, the concentrations of which ranged from 17 to 1002 µg/kg. The presence of so many antibacterial substances is the result of the input used for biogas production, which, according to the manufacturer’s declaration, was corn silage, pig slurry, chicken manure, meat and bone meal (pulp), beef mulch, waste from feed production, waste from on-site sewage treatment plants, raw materials and products unsuitable for consumption, and feed mixtures. Most of the substrates used for biogas production could therefore contain antibiotics and quinolones, which were not degraded despite the processing process, but the presence of as many as seven different antibacterial substances in one sample shows how often and with what intensity these compounds are used in farm animals.

In the case of the positive results obtained for samples of pig slurry and slurry from dairy cows, the presence of antibiotics was found in concentrations higher than in organic liquid fertilizers but in concentrations lower than those described in solid natural fertilizers in previous works by Patyra et al. [8]. The slurry samples contained oxytetracycline and its epimeric form, as well as doxycycline and tiamulin at concentrations ranging from 262 µg/kg to nearly 15,000 µg/kg [8]. Lower concentrations of antibiotics in the slurry may be due to the fact that slurry is a mixture of feces, urine, and water used to clean livestock premises. In addition, every farmer keeping animals and collecting liquid manure must have appropriate tanks to store it for several months. As a result, the antibiotics present in the slurry are diluted, and their storage for several months leads to the partial degradation of antibiotics by microorganisms, changes in temperature and pH, and other processes occurring during the storage of liquid feces.

We did not find antibiotic residues in commercial organic liquid fertilizers produced with animal by-products, such as biohumus, liquid horse manure, cattle manure extract, or organic fertilizers intended for use in the cultivation of vegetables, balcony plants, and green plants. The lack of residues in these fertilizers may be due to the use of animal excrement from organic farming or processing processes, e.g., composting and the use of California earthworms.

In a study conducted by Widyasari-Mehhta et al., research on the presence of antibiotics in slurry samples and post-fermentation sludge was carried out on 21 farms located in Lower Saxony, Germany, engaged in pig breeding, and on 9 farms with their own biogas plants. The authors analyzed 19 selected compounds belonging to the group of sulfonamides, tetracyclines, fluoroquinolones, macrolides, and pleuromutilins. Research has shown that pig slurry contains up to several hundred milligrams of antibiotics per kilogram. The authors determined tetracycline in pig slurry in the concentration range from 1.5 to 300 mg/kg dry weight (DW). Then, the highest concentrations were determined for chlortetracycline and doxycycline, the amount of which in the dry matter of slurry ranged from 1.7 to 55 mg/kg DW and from 5 to 166 mg/kg DW, respectively. Additionally, the authors found oxytetracycline, sulfadiazine, sulfamethazine, sulfadimethoxine, and trimethoprim in the tested slurry samples at concentrations ranging from 0.5 mg/kg DW to above 200 mg/kg DW [10]. The results of other authors’ studies conducted in China and Austria also showed very high concentrations of tetracycline antibiotics in pig slurry, which amounted to 764 mg/kg DW for chlortetracycline and 770 mg of oxytetracycline kg DW [28,29]. Our research also confirmed that doxycycline is most frequently found in pig slurry, and in slurry from dairy cows, we detected oxytetracycline, but the concentrations we determined were much lower. This was due to the fact that our work included the determination of antibiotics in a liquid sample, not in the dry mass of slurry. This approach resulted from the fact that liquid slurry is used on agricultural fields, not its dry fraction.

Vidyasari-Mehta et al., in addition to analyzing pig slurry, also examined post-fermentation sludge from biogas plants. In five biogas plants from which samples were taken, pig manure and corn silage were used as input for biogas production. In the next four biogas plants, the input for biogas production was, among others, liquid cattle slurry, dry poultry manure, and whole-plant silage. The materials used to produce biogas came from different farms, and the biogas plants differed in construction, size, and the amount of electricity produced. In the dry mass of post-fermentation sludge from biogas plants, Vidyasari-Mehta et al. detected the presence of doxycycline at concentrations above 10 mg/kg, as well as sulfadiazine, chlortetracycline, tetracycline, and enrofloxacin at concentrations ranging from 0.2 mg/kg to 2.1 mg/kg DW [10]. The results obtained by Widyasari-Mehta and Ratsak et al., Spielmayer et al., and Gans et al. confirmed that methane fermentation processes in biogas plants do not lead to the complete degradation of antibiotics present in the material used for biogas production [10,13,14,28]. The results obtained by other authors coincide with those obtained in this work. Because we also confirmed the presence of many antibiotics in post-fermentation sediments, the most frequently found antibiotics were enrofloxacin and doxycycline. The concentrations we determined are lower than those indicated in other publications, but this is due to the fact that our tests were conducted using liquid digestate samples and not the dry matter obtained from the digestate. Our assumption is that post-fermentation sludge is used in liquid form as organic fertilizers for agricultural fields and permanent grasslands. Therefore, the assessment of the content of antibiotics present in them should be carried out in the same form as that used in agricultural fields. The estimation of concentrations in a liquid sample will be more reliable for conducting further research related to, among others, environmental risk assessments after introducing natural and organic liquid fertilizers into the natural environment. The results for the tested samples of natural and organic liquid fertilizers in which antibiotics were found are presented in Table 2.

## 3. Materials and Methods

### 3.1. Sample Collection

Manure samples from eight pigs and one cattle manure sample were collected from animal farms in Lubelskie Voivodship in Poland. Fifty-three samples of organic liquid fertilizers such as fermentation sludge from biogas plants; sludge from composting and biocomposting plants and from the processing plant; and commercial organic liquid fertilizers produced with animal by-products, such as biohumus, liquid horse manure, cattle manure extract or organic fertilizers intended for use in the cultivation of vegetables, balcony plants, and green plants were obtained from biogas plants or composting plants located in Poland and purchased from gardening stores. The samples were transported to the laboratory, and before analyzing the samples, they were stored at −20 °C.

### 3.2. Standards, Reagents, and Chemicals

HPLC-grade acetonitrile and methanol were purchased from J.T. Baker (Deventer, The Netherlands). Citric acid and formic acid (purity > 99% for analysis) were obtained from Acros Organics (Geel, Belgium). Disodium ethylenediamine tetraacetate (Na_2_EDTA) was obtained from Sigma-Aldrich (CA, MO, USA), and disodium hydrogen phosphate was obtained from Chempur (Piekary Śląskie, Poland). Water was purified using a Milli-Q water system from Millipore (Billerica, MA, USA). Strata-X cartridges (200 mg/6 mL) were purchased from Phenomenex (Torrance, CA, USA).

All antibacterial substances and internal standards (ISs) were purchased from Dr. Ehrenstorfer Gmbh (Augsburg, Germany). A panel of twenty-one antimicrobials belonging to six classes was used to develop the method, including pleuromutilin (VAL and TIAM), fluoroquinolones (ENR, CIP, FLU, SAR, and norfloxacin (NOR; IS)), sulfonamides (SMZ, SDZ, SMR, SGD, SMX, and sulfadiazine-^13^C_6_ (SDZ-^13^C_6_; IS)), tetracyclines (CTC, epi-CTC, DC, OXT, epi-OXT, TC, and demeclocycline (DMC; IS)), macrolides (SPIR), TYL) and erythromycin (ERM; IS), diaminopyrimidines (TRIM), and lincosamides (LINCO and lincomycin-d_3_ (LINCO-d_3_; IS)).

Primary standard solutions of analytes and internal standards were prepared at 1 mg/mL concentrations in methanol. As a result of limited solubility in methanol, stock standards of SDZ, SDZ-^13^C_6,_ FLU, and NOR were prepared in acetonitrile, and CIP was dissolved in a mixture of methanol and 1M sodium hydroxide (99:1; *v*/*v*) and stored at −20 °C. Working solutions and internal standards were prepared using stock solutions diluted with methanol at a concentration of 10 µg/mL, which was stored at −20 °C.

### 3.3. Extraction Procedure

The liquid manure, digestate samples, and commercial organic liquid fertilizers samples were analyzed for the 21 selected antibacterial substances from 6 different chemical classes. The pH of natural liquid fertilizers is approximately 7.0 to 9.0. To enable the isolation of all antibacterial substances that were selected for testing, natural and organic liquid fertilizers were acidified to pH ≈ 4 with 85% orthophosphoric acid before extraction. Then, 5 g of natural and organic liquid fertilizers were weighed into 50 mL centrifuge tubes. Antibiotics from samples were extracted using a mixture of acetonitrile, methanol, and McIlvaine–Na_2_EDTA buffer with pH = 4, in the proportion of 3.75:1.25:3 *v*/*v*/*v*. The samples were shaken and centrifuged. Solid-phase extraction on Strata-X cartridges (6 mL, 200 mg) was used to purify the extract. Due to the fact that these are polymeric cartridges with a reversed phase, the obtained extract, which contained 60% of organic solutions (acetonitrile and methanol), required dilution so that the antibiotics could be retained on the SPE cartridge filling. Therefore, 6 mL of the extract was transferred to a new centrifuge tube, and 25 mL of water was added. The cartridges were conditioned with methanol and water, and then the sample was dosed, and the cartridges were washed with water. The cartridges were dried under vacuum for 10 min, and the antibiotics were eluted with methanol. The obtained extract was dried in a stream of nitrogen, and the precipitate was dissolved in 500 µL of 0.1% formic acid in water and additionally filtered through a PVDF syringe filter. The internal standards mixture was used before extraction (40 µL).

### 3.4. Antibiotic Detection by LC-MS/MS Analysis

The quantification of antibiotics was performed by UHPLC-MS/MS consisting of an Exion LC with a SCIEX Triple Quad 5500+ System (SCIEX, Framingham, MA, USA). An LC analysis was performed using an Exion LC equipped with a thermostated autosampler, degasser, and binary pump, and connected in series to an AB Sciex 5500+ QTRAP mass spectrometer equipped with a Turbo Ion Spray source that was operated in the positive mode. The curtain gas, ion source gas 1, ion source gas 2, and collision gas were set at 30 psi, 40 psi, 40 psi, and “medium” instrument units, respectively, and the ion spray voltage and source temperature were set at 4500 V and 400 °C, respectively. Chromatographic separation was achieved with a Kinetex C18 column (2.1 mm × 75 mm; 2.6 µm); the column thermostat was set at 35 °C, and the flow rate of the mobile phase was 0.25 mL/min. The mobile phase consisted of HPLC-grade water with 0.1% formic acid as eluent A and acetonitrile with 0.1% formic acid as eluent B. The gradient (%B) was as follows: 0–2 min 5% B; 2–10 min 5–15% B; 10–12 min 15–20% B; 12–15 min 20–50% B; 15–16 min 50–70% B; 16–17 min 70–100% B; 17–18 min 100–5% B; and 18–21 min 5% B. The target compounds were analyzed in multiple reaction monitoring (MRM) mode in the positive ionization mode (ESI +) for all antibiotics, monitoring two transitions between the precursor ion and the most abundant fragment ions for each compound.

### 3.5. Validation of the Method

The validation of the developed method was carried out in accordance with the guidelines contained in the EU Commission Implementing Regulation 2021/808 [27]. The following validation parameters were determined: repeatability, intralaboratory reproducibility, linearity, recovery, the limit of quantification and limit of detection, decision limit (CCα), detection capability (CCβ), and the selectivity and uncertainty of the method. In order to assess repeatability and intralaboratory reproducibility, a set of six replicates at three concentration levels (10, 50, and 500 µg/kg and 20, 100, and 750 µg/kg for ciprofloxacin, enrofloxacin, and sarafloxacin) was used. Intralaboratory reproducibility was performed by spiking two other sets of blank slurry samples at the same concentrations for repeatability and analyzing them on different days. The selectivity of the method was checked by analyzing 20 blank samples to verify the absence of potentially interfering endogenous compounds. CCα and CCβ were calculated from the standard curve according to ISO 11843-1:1997 [30]. The limit of detection and limit of quantification were evaluated based on the signal-to-noise ratio (3 for LOD and 10 for LOQ). The linearity was determined with a matrix calibration curve over a range of seven concentrations (0, 10, 25, 50, 100, 250, and 500 µg/kg, and for CIP, ENR, and SAR, 0, 20, 50, 100, 250, 500, and 750 µg/kg). Measurement uncertainty is a parameter related to the measurement result, characterizing the dispersion of values that can reasonably be attributed to the measured quantity. The total uncertainty was expressed as the combined standard uncertainty (uc), and the expanded uncertainty (U) was taken as the product of the combined standard uncertainty and the coverage factor k = 2.

## 4. Conclusions

The presented work describes the optimization and validation of the LC-MS/MS method for the analysis of 21 antibacterial substances in natural and organic liquid fertilizers. As a result of the validation, satisfactory results were obtained for all antibacterial substances. Moreover, the analysis of 62 samples of liquid fertilizers showed the presence of a wide range of antibacterial substances. The presence of antibiotics from the tetracycline group (doxycycline and oxytetracycline) was found in pig slurry and slurry from dairy cows. The presence of antibiotics was also found in the analyzed post-fermentation sludge from the biogas plant. The most frequently found antibiotics were enrofloxacin and doxycycline. As many as seven different antibiotics were determined in one sample of post-fermentation sludge. The obtained results confirmed that the technological processes occurring during biogas production do not lead to the complete degradation of antibiotics present in the material used. Due to the fact that antibiotics are used on a large scale in farm animals, the use of their excrements and post-fermentation sludge from biogas plants should be monitored in terms of the presence and content of antibacterial substances before their use on agricultural fields and permanent grasslands in order to protect the natural environment and do not cause the risk of developing drug-resistant bacteria.

## Figures and Tables

**Table 1:** Validation results of the LC-MS/MS method for determining antibiotics in liquid fertilizers.

Validation Parameters	Analyte										
	OXT	epi-OXT	TC	CTC	epi-CTC	DC	CIP *	ENR *	SAR *	FLU	
Selectivity	No interference										
LOD (µg/kg)	4.4	5.1	3.1	4.0	4.3	6.2	* 12.0 *	* 13.1 *	* 10.2 *	3.1	
LOQ (µg/kg)	7.5	7.2	5.1	6.0	7.4	8.5	* 15.6 *	* 17.8 *	* 18.2 *	6.0	
CCα (µg/kg)	12.3	15.2	11.3	10.2	14.8	15.4	* 25.8 *	* 22.2 *	* 28.0 *	12.2	
CCβ (µg/kg)	15.6	18.1	15.6	13.4	17.7	21.0	* 36.3 *	* 31.0 *	* 37.6 *	17.4	
Repeatability (CV %)											
10/***20*** * µg/kg	8.0	10.1	6.4	12.0	12.0	15.6	* 16.0 *	* 18.0 *	* 16.8 *	10.3	
50/***100*** * µg/kg	8.1	9.2	7.2	10.1	11.0	14.8	* 14.9 *	* 16.8 *	* 9.8 *	8.8	
500/***750*** * µg/kg	5.6	10.3	5.3	9.3	8.0	17.6	* 15.1 *	* 17.0 *	* 9.0 *	9.4	
Reproducibility (CV %)											
10/***20*** * µg/kg	9.1	14.3	12.7	15.5	18.2	16.8	* 17.8 *	* 18.1 *	* 15.6 *	13.1	
50/***100*** * µg/kg	11.0	12.1	9.5	12.5	11.8	17.0	* 13.8 *	* 19.0 *	* 16.7 *	15.0	
500/***750*** * µg/kg	9.4	14.7	7.6	10.1	9.5	19.2	* 14.9 *	* 17.6 *	* 12.3 *	13.2	
Recovery (%)											
10/20* µg/kg	115.4	120.4	98.6	112.3	100.4	117.0	* 96.4 *	* 113.9 *	* 122.1 *	112.2	
50/***100*** * µg/kg	98.6	98.5	101.2	101.1	101.3	97.5	* 99.5 *	* 98.5 *	* 97.7 *	95.5	
500/***750*** * µg/kg	102.1	114.6	114.2	98.7	111.4	96.7	* 102.1 *	* 104.7 *	* 93.2 *	88.6	
Uncertainty (U %)											
10/***20*** * µg/kg	25.1	28.1	24.6	31.0	26.6	35.0	* 33.0 *	* 36.0 *	* 31.0 *	25.2	
50/***100*** * µg/kg	22.3	25.2	18.0	25.2	25.0	34.3	* 28.1 *	* 35.2 *	* 31.2 *	27.3	
500/***750*** * µg/kg	21.9	26.6	15.6	21.0	20.3	35.6	* 28.0 *	* 30.9 *	* 25.5 *	26.1	
**Validation parameters**	**Analyte**										
	**SGD**	**SDZ**	**SMR**	**SMZ**	**SMX**	**TRIM**	**LINKO**	**TIAM**	**TYL**	**SPIR**	**VAL**
Selectivity	No interference										
LOD (µg/kg)	6.3	3.0	3.4	3.1	4.0	3.3	5.9	2.9	5.8	4.2	5.8
LOQ (µg/kg)	8.9	5.4	6.5	6.3	7.6	6.2	7.6	5.2	8.7	8.2	7.8
CCα (µg/kg)	16.7	11.1	17.7	12.1	10.3	12.1	13.4	11.7	17.7	13.8	14.7
CCβ (µg/kg)	25.3	17.2	23.8	16.1	15.4	18.4	18.5	14.5	26.4	19.4	20.1
Repeatability (CV %)											
10/***20*** * µg/kg	17.1	10.3	16.1	14.1	14.5	9.8	12.1	11.4	16.6	11.0	12.3
50/***100*** * µg/kg	16.0	11.4	12.5	13.4	11.3	9.4	13.5	10.8	17.1	10.6	15.0
500/***750*** * µg/kg	12.0	5.7	11.9	7.7	9.8	11.7	12.1	13.0	13.4	8.5	11.4
Reproducibility (CV %)											
10/***20*** * µg/kg	20.1	10.8	17.8	12.9	17.1	12.8	14.5	17.5	17.5	13.0	15.6
50/***100*** * µg/kg	15.4	17.8	13.5	15.4	10.4	11.6	13.9	14.3	16.7	12.1	14.5
500/***750*** * µg/kg	10.0	12.6	11.2	11.2	10.2	10.1	11.0	11.9	13.8	10.5	12.1
Recovery (%)											
10/***20*** * µg/kg	100.2	110.2	113.2	102.2	101.2	111.2	97.3	106.7	94.4	105.2	112.8
50/***100*** * µg/kg	91.3	112.0	98.7	104.3	94.3	110.3	98.5	103.4	98.7	100.7	107.6
500/***750*** * µg/kg	97.7	93.3	99.5	98.3	97.8	109.4	101.2	98.3	103.8	98.8	102.2
Uncertainty (U %)											
10/***20*** * µg/kg	30.2	28.6	29.4	28.0	31.0	25.0	26.7	26.0	33.5	26.0	25.4
50/***100*** * µg/kg	30.6	25.1	26.3	26.3	24.5	22.7	25.1	25.1	30.7	22.2	27.8
500/***750*** * µg/kg	19.5	24.4	22.1	22.8	18.5	19.6	23.5	23.0	28.9	19.5	23.1

OXT—oxytetracycline, epi-OXT—epi-oxytetracycline, TC—tetracycline, CTC—chlortetracycline, DC—doxycycline, CIP—ciprofloxacin, ENR—enrofloxacin, SAR—sarafloxacin, FLU—flumequin, SGD—sulfaguanidin, SDZ—sulfadiazin, SMR—sulfamerazin, SMZ—sulfamethazin, SMX—sulfamethoxazol, TRIM—trimethoprim, LINCO—lincomycin, TIAM—tiamulin, TYL—tylosin, SPIR—spiramycin, VAL—valnemulin. * Levels marked with an asterisk apply only to ciprofloxacin, enrofloxacin, and sarafloxacin.

**Table 2:** Results for positive samples of natural and organic liquid fertilizers.

No.	Analyte [µg/kg]							
	Sample	DC	OXT+ epi-OXT	CIP	ENR	TIAM	TYL	LINCO
** Organic liquid fertilizers produced from animal by-products **								
1.	Post-fermentation sludge from a biogas plant					148.6		
2.	Post-fermentation sludge from a biogas plant				45.6			
3.	Post-fermentation sludge from a biogas plant	89.2						
4.	Post-fermentation sludge from a biogas plant				135.3			
5.	Post-fermentation sludge from a biogas plant				166.7	54.3		63.6
6.	Post-fermentation sludge from a biogas plant			24.4	43.5		27.3	
7.	Post-fermentation sludge from a biogas plant			61.9	456.3			
8.	Post-fermentation sludge from a biogas plant	82.7						
9.	Post-fermentation sludge from a biogas plant				32.3			
10.	Post-fermentation sludge from a biogas plant				13.8			
11.	Post-fermentation sludge from a biogas plant	1002.0	23.0	38.0	216	426.0	17.0	192.0
** Liquid manure **								
12.	Slurry from milk cows		1500.0 + 324.6					
13.	Pig slurry	14,810				262.0		
14.	Pig slurry	5640.7						

## Data Availability

The data presented in this study are available on request from the corresponding authors.
